# Native Hawaiian and Pacific Islander community-led survey on COVID-19 and willingness to participate in clinical trials

**DOI:** 10.1186/s12889-026-26393-6

**Published:** 2026-02-11

**Authors:** Mona AuYoung, Keni Lasitani, Tana Lepule, Raynald Samoa, Nia Aitaoto

**Affiliations:** 1 David Geffen School of Medicine, University of California, Los Angeles, Los Angeles, CA USA; 2 Scripps Health, San Diego, CA USA; 3 Pacific Islander Collective San Diego, San Diego, CA USA; 4 University of California San Diego School of Medicine, San Diego, CA USA; 5 City of Hope National Medical Center, Duarte, CA USA; 6 Pacific Islander Center of Primary Care Excellence, San Leandro, CA USA

**Keywords:** Native hawaiians and pacific islanders, Community-based organizations, Diversity, Clinical trials, Trust

## Abstract

**Background:**

Native Hawaiians and Pacific Islanders (NHPIs) have historically been erased from public health and research data due to smaller population size and mis-categorization. This became a bigger problem during the COVID-19 pandemic where NHPIs were disproportionately impacted, yet this was largely hidden because NHPI data were grouped with Asian Americans or aggregated into the Other category. This study assesses the impact of COVID-19 on the NHPI community as well as their willingness to participate in clinical trials.

**Methods:**

This cross-sectional study took place between April 2021 and January 2022. Individuals in San Diego County who identified as NHPI were eligible to complete the survey on behalf of their households. The primary outcomes are willingness to get vaccinated and to participate in clinical trials as measured by Likert scales.

**Results:**

Of the 130 households (representing 605 individuals) that responded to the survey, the majority were Samoan (53%) and Chamorro (30%) with an average household size of 4.6 individuals. Most (88%) households had one or more essential workers. The biggest COVID-19 challenges for respondents were paying for rent or food, caring for sick relatives, and dealing with lost jobs. Nearly half of households relied on support from community-based organizations, especially for meals/groceries, emotional/spiritual/mental health support and COVID-19 resources/vaccines. There was a stark contrast between willingness to get vaccinated (67% had gotten their second vaccine dose) and willingness to participate in a clinical trial (56% not at all likely to participate). This paralleled the difference in having a great deal of trust in COVID-19 information from doctors (69%) compared to researchers (37%).

**Conclusions:**

This paper describes the gap between policy and practice, specifically a mismatch of available resources (for example, personal protective equipment and free COVID-19 tests from workplaces and the federal government) to those at disproportionate risk of exposure and severe outcomes during a pandemic. It also discusses the benefits of having local community-based organizations to help fill gaps in the public health system. Future research is needed to explore the reasons for lower levels of trust in researchers and less willingness to participate in clinical trials, which has implications for equity in research.

**Supplementary Information:**

The online version contains supplementary material available at 10.1186/s12889-026-26393-6.

## Background

Native Hawaiians and Pacific Islanders (NHPIs) are one of the fasting growing populations in the United States, but the exclusion of NHPIs from datasets continues to be a problem in research, health care, and public health. This is despite federal standards for data collection established in 1997 (with categories that distinguished NHPIs from Asians) and updated in 2024 [1–4]. NHPIs continue to be grouped incorrectly with Asians or Other, rendering the entire community invisible when it comes to reporting research findings, population health outcomes, and particularly during a global public health emergency. This is despite having unique histories and health risk profiles that require precise data that can help with designing appropriate interventions or allocating resources [1, 2, 5]. The COVID-19 pandemic disproportionately impacted NHPIs living in the continental United States [5–8]. During the beginning of the pandemic, safety guidelines to stay home and keep a distance from others were issued across the country to try to reduce transmission of the virus. However, this was not an option for essential workers or members of large households; NHPIs are overrepresented in essential work (in Hawai’i, 24% of NHs are essential workers) and many live in large, multigenerational households and/or densely populated neighborhoods [5, 7, 9]. Furthermore, scientists quickly identified common risk factors for severe cases of COVID-19, including being of older age, being immunocompromised, or having underlying conditions such as diabetes, obesity, or asthma; a disproportionately high percentage of NHPIs experience those health conditions [7, 8, 10]. Race/ethnicity was also identified as a risk factor, with the recognition that this is likely related to socioeconomic factors such as lack of access to health care or lack of paid sick leave. Once again, NHPIs carried a disproportionate burden of these risk factors [9], yet were not included in news stories or public health data about which communities were being affected by COVID-19.

Despite the National Institutes of Health (NIH)’s Revitalization Act of 1993, which mandated the inclusion of minorities (including women, older adults, and racial/ethnic groups) in all NIH-funded research, there continues to be a lack of clinical trials that include diverse participants [11, 12]. The issue became even more urgent during the pandemic, as scientists around the world raced to develop vaccines and treatments for COVID-19. In September 2020, Moderna slowed down enrollment into their vaccine clinical trial in order to increase the representation of minority populations which had been most affected by COVID-19 [13]. In November 2020, the United States Food and Drug Administration (FDA) released a statement to accompany their guidelines to encourage more diverse participation in clinical trials, especially for the development of medical products [14]. However, while studies were paying more attention to the racial/ethnic diversity of their participants, few were disaggregating the data for NHPIs (for both data collection and analysis). For example, one study found that NHPI COVID-19 patients were hospitalized at younger ages than other racial/ethnic groups, in contrast to public guidance to protect older adults [15]. Another recent paper describes strategies to increase community access to relevant data for smaller populations like NHPIs [16]. This paper describes the impact of COVID-19 on NHPIs, their trust in various information sources, willingness to participate in clinical trials and intent to get the COVID-19 vaccine.

## Methods

Community leaders across the country formed the National Pacific Islander COVID-19 Response Team (NPICRT) with NHPI researchers, health experts, community leaders, and advocates to provide the emergency response that was missing at local and federal levels for the NHPI community. The NPICRT designed the NHPI household survey (see supplemental file) to assess the impact of COVID-19 on NHPI families living in the continental U.S, asking for one response per household. This paper focuses on responses from San Diego County because it has the third highest NHPI population in the United States and at the time of data collection, had three active COVID-19 vaccine clinical trials along with clinical trials for treatments. Informed consent to participate was deemed unnecessary because this was part of NPICRT’s operations during this public health emergency, but Institutional Review Board (IRB) approval was obtained through the Association of Asian Pacific Community Health Organizations (AAPCHO) for this secondary data analysis.

### Measures

#### Demographics

The questionnaire asked about household racial groups (Polynesian, Micronesian, Melanesian, other), with options to name specific ethnic groups within each category, as well as the number of family members within specific age groups (e.g., 0–4 years, 5–12 years, etc.).

#### Household size

Respondents were asked for overall household size, defined as the number of people living in the house. 

#### Language

Respondents were asked if at least one adult in the household spoke English, how well they spoke English, and preferred language to receive information on health and other services.

#### Occupation

A group of questions asked about occupation, including one question about the number of household members employed in each of several essential occupations (e.g., healthcare or health services, food preparation or service, transit/transportation worker, etc.). Respondents were also asked if any household members were continuing to leave the house to go to work and if their employer provided personal protective equipment (i.e., mask, gloves) to prevent viral transmission. There was also a question asking if any household members had been laid off or furloughed due to COVID-19.

#### Healthcare access

Respondents were asked how many household members had a primary care doctor, the last time they saw a health care provider for a check-up (not when sick), usual source of health care, health insurance status, and health literacy.

#### Health status

The questionnaire had a checklist of common health conditions (e.g., high blood pressure, obesity, diabetes, asthma, etc.) where they could report the health status of up to five household members.

#### COVID-19

Respondents were asked if anyone in the household had tested positive for COVID-19, how they found out, whether they were symptomatic, if they received treatment, if they were hospitalized, and the outcomes (including age at death, if applicable).

#### Resources

There was a checklist of social and direct services that respondents could mark if they needed help in those areas due to COVID-19 (e.g., official information in my primary language, food, utilities, mental health care, small business support, etc.). Follow up questions asked for the top three challenges they were facing and whether any community-based organizations (CBOs) had provided any support. The survey defined CBOs as local organizations that worked to address community needs, including social service agencies, nonprofit organizations, and formal and informal community groups, like neighborhood groups or recreational clubs.

#### COVID-19 clinical crials

Respondents were asked if anyone in their household had participated in a COVID-19 clinical trial (vaccine or treatment), whether they knew how to sign up for a clinical trial, and also how likely they would be to sign up for a clinical trial, a measure adapted from Yang et al. [17].

#### COVID-19 Vaccines

A series of questions about the COVID-19 vaccines included one about concerns or questions (respondents could mark a checklist or fill in their own response), whether anyone in the household had received a vaccine, whether anyone knew how to make an appointment to get a vaccine, and how likely household members would be to get vaccinated once eligible [18]. 

#### Trust in COVID-19 information

Respondents were also asked how much they trusted various sources (e.g., health care providers, faith leaders, news, social media) to provide correct information about COVID-19. The trusted sources of information about COVID-19 were adapted from the CEAL program’s use of the DMACS COVID-19 Survey [19]. 

The NPICRT disseminated the survey to team members in California, but with vaccine trials underway, members of the San Diego team first modified the questionnaire to include questions about participation in clinical trials and vaccine uptake. Members of the San Diego team included an NHPI community leader and a NHPI medical student (now resident) working with a local vaccine trial. The team used multiple channels to disseminate the survey and rely on snowball sampling through emails to local churches, community-based organizations, large cultural events and community vaccination events. Adults that identified as NHPI were eligible to complete the survey on behalf of their households. The questionnaires were programmed into SurveyMonkey [20] for community members to take the survey by clicking on a link on the study tablet or scanning a QR code on the study tablet or their own cell phone. They could also take a flyer with the survey link and QR code to complete the survey at home. The data were analyzed with Stata/SE 18.0 [21]. 

## Results

### Characteristics of responding households

Between April 2021 and January 2022, we collected responses from 130 NHPI households (representing 605 individuals). The households were located all over San Diego County, although 45.0% were from the central part of the county, where there is a significant NHPI population. The majority of respondents were Samoan (53.1%) and Chamorro (30.0%) with an average household size of 4.7 (ranging from one to 16) individuals (see Table 1). Out of the total number of individuals across responding households, most (27.3%) were young adults ages 18–34, 23.3% were under age 18 and only 6.9% were age 65 or older. About a quarter of households have at least one U.S. Veteran and a majority (73.1%) of households reported that they attend church. Even though 99.2% of households reported that at least one adult spoke English and 93.8% reported that this person spoke English very well, 13.8% of households still reported a preference for receiving health information in an NHPI language such as Samoan, Chamorro, or Tongan.

**Table 1:** Demographics of respondents (*N* = 130 households)

Household Characteristics	
Ethnic group	
*Native Hawaiian*	12 (10.0%)
*Samoan*	69 (53.1%)
*Tongan*	4 (3.1%)
*Chamorro*	39 (30.0%)
*Chuukese*	2 (1.5%)
*Fijian*	2 (1.5%)
*Missing*	1 (0.8%)
**Residence**	
*East County*	31 (24.0%)
*South County*	32 (24.8%)
*Central County*	58 (45.0%)
*North County*	8 (6.2%)
**Number of household members by age group** ^a^	
*0–17 years*	141 (23.3%)
*18–34 years*	165 (27.3%)
*35–44 years*	98 (16.2%)
*45–54 years*	90 (14.9%)
*55–64 years*	69 (11.4%)
*65 years+*	42 (6.9%)
Average household size (range)	4.7 (1–16)
At least one active duty military in household	8 (6.2%)
At least one U.S. Veteran in household	33 (25.6%)
Attend church	95 (73.6%)
Employment	
At least one household member has had to leave house to work (during pandemic)	113 (87.6%)
Access to PPE from employer	82 (63.6%)
At least one household member furloughed, laid off, or reduced hours due to pandemic	70 (54.3%)
Health	
Household members have a regular place to go when sick	102 (78.5%)
Never need help with reading health information	109 (83.9%)
At least one household member has a chronic health condition^a^	108 (83.1%)
*High blood pressure*	98 (16.2%)
*Obesity*	121 (20.0%)
*Diabetes*	57 (9.4%)
*Lung disease (not asthma)*	13 (2.1%)
*Asthma*	37 (6.1%)
*Heart disease*	23 (3.8%)
*Kidney disease*	23 (3.8%)
*Liver disease*	5 (0.8%)
At least one household member is immunocompromised^a^	40 (30.8%)
*Weakened immune system (not cancer)*	38 (6.3%)
*Cancer*	12 (2.0%)
At least one household member tested positive for COVID-19	43 (33.1%)

^*a*^These numbers refer to the total number of individuals (*N* = 605) across responding households (*N* = 130)

### Impact of COVID-19

Most households (87.6%) reported at having least one essential worker (as many as six in one household), with the most common occupations in healthcare or health services, community and social services, and school or childcare. In addition, 83.1% of households reported at least one household member with a chronic disease (e.g., heart disease, diabetes) and 30.8% reported at least one immunocompromised household member. This means many household members faced both greater risk of exposure and potential for severe outcomes (see Table 1). However, only 63.6% of total households reported the ability to access personal protective equipment (PPE) from their employers. Many households (46.9%) reported using support from community-based organizations: meal pickup/delivery, emotional/spiritual/mental health support, COVID-19 resources/vaccines, financial assistance, school-related assistance, and childcare. This matched the three biggest concerns reported: paying for rent, food, and other necessities (21.0%), caring for sick relatives (9.3%), and losing their job (8.9%). 

### Willingness to get vaccinated and participate in clinical trials

**Fig. 1:**
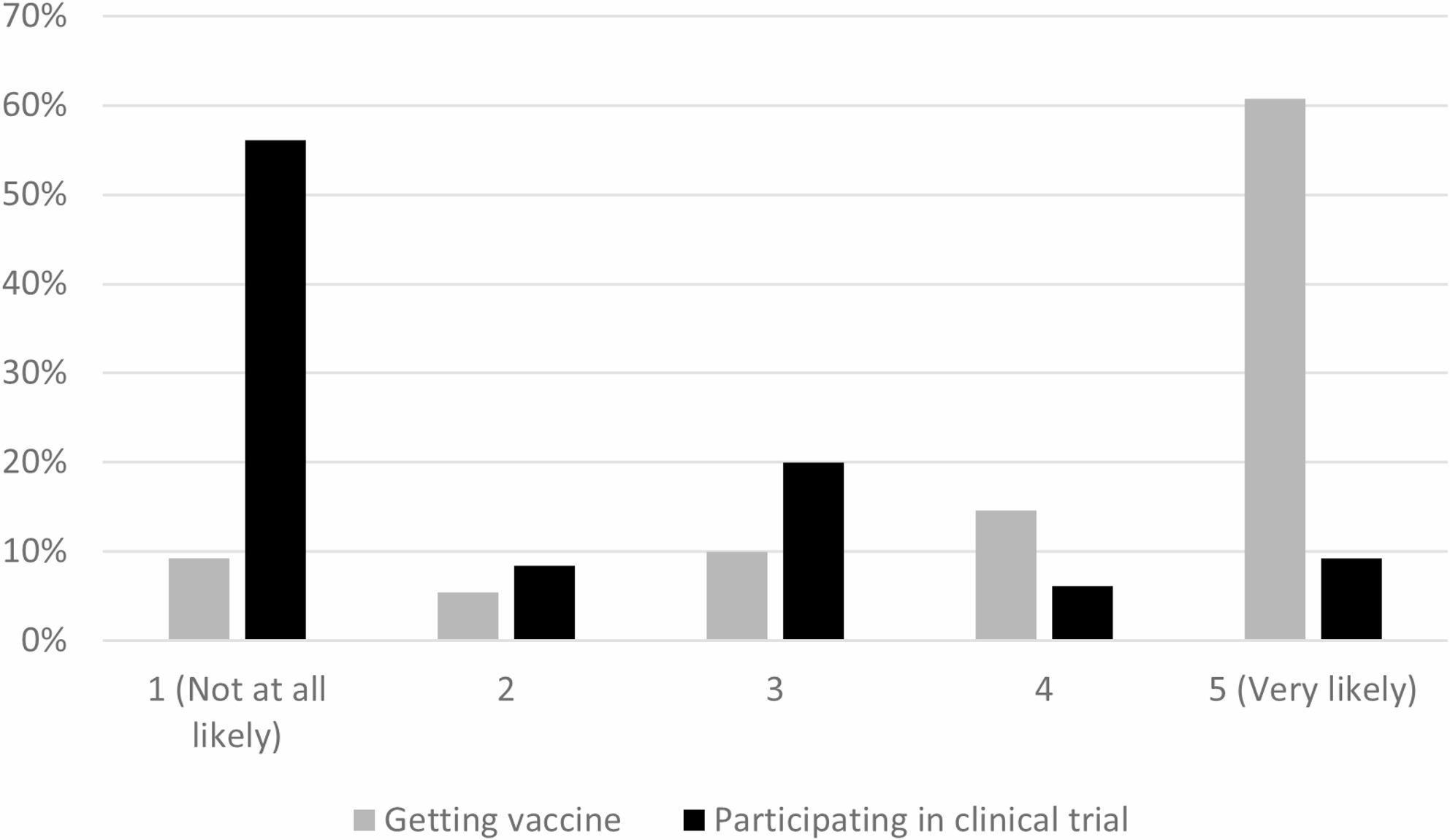
Comparison of Likelihood of Getting COVID-19 Vaccine or Participating in Clinical Trial (N=130)

**Fig. 2:**
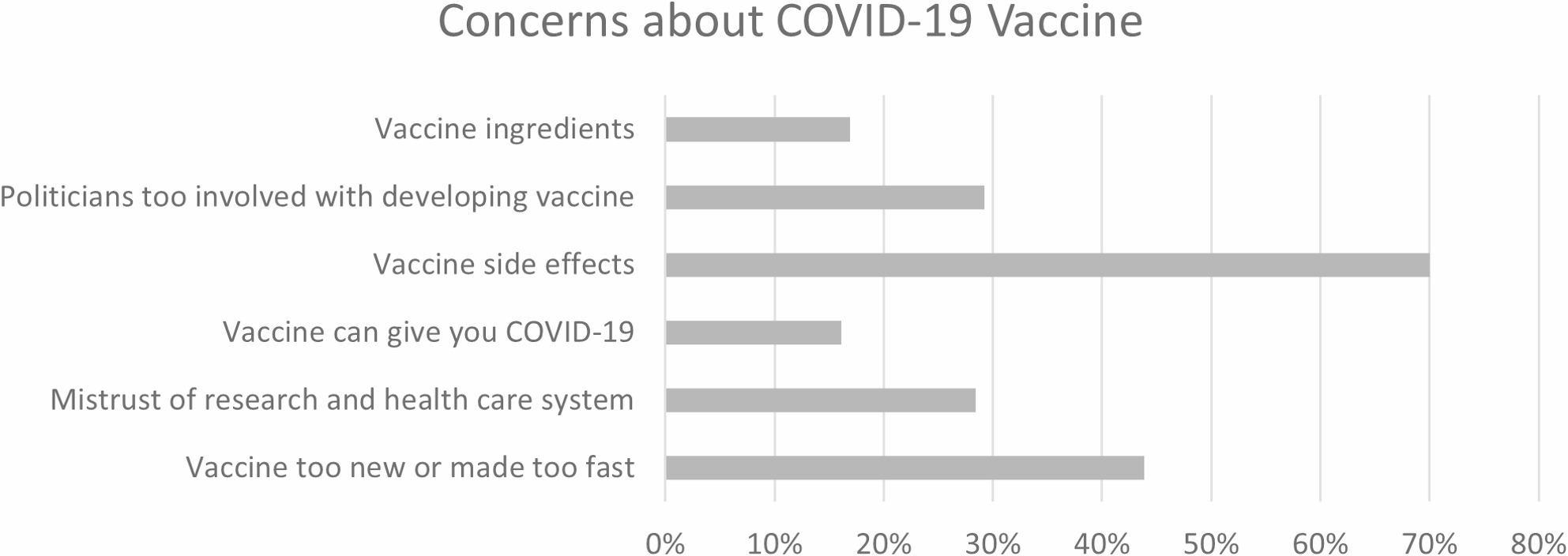
Concerns about COVID-19 Vaccine among Households with any Vaccine Concerns (N=118)

San Diego had three different vaccine clinical trials in progress at the time and additional clinical trials for COVID-19 treatments, but 56.2% of households were not likely at all to participate in COVID-19 clinical trials for vaccines or treatments (see Fig. 1). Only 21.5% knew how to sign up for clinical trials and 5.4% had someone who had signed up to participate in a clinical trial. In contrast, 60.8% of households reported that members of their household were very likely to get a COVID-19 vaccine. Among respondents, 66.9% reported having received their second vaccine dose, although this may be underreporting those willing to get the second dose, but were not yet scheduled due to timing between doses. The most common concern about the COVID-19 vaccine was side effects (70.0%), followed by how quickly the vaccine was developed or that it was too new (43.9%) (see Fig. 2).

### Trust in sources of COVID-19 information

**Fig. 3:**
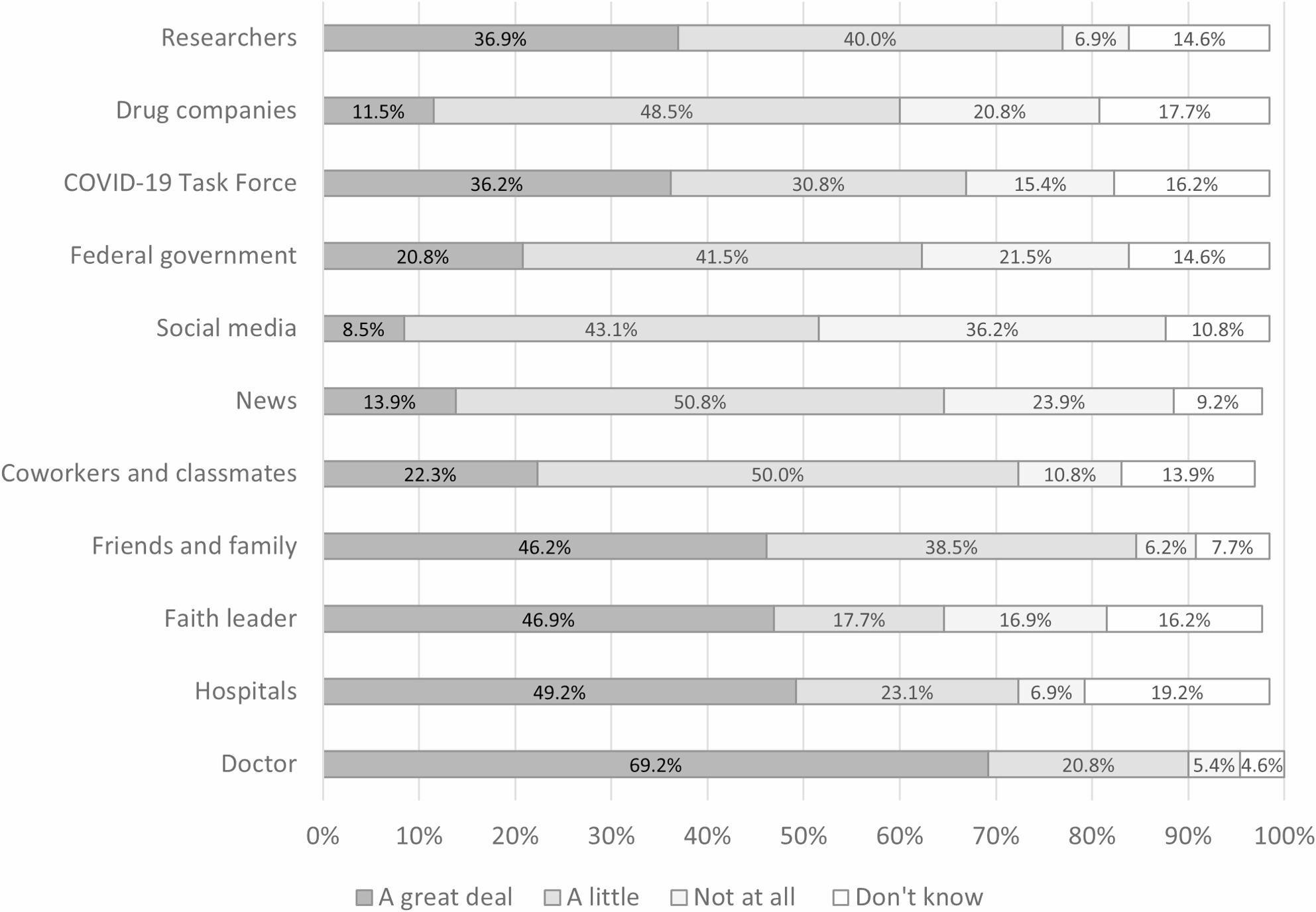
Degree of trust in sources to provide correct information about COVID-19 (N=130)^a^

^a^Due to missing data, the denominator is *N* = 128 for researchers, drug companies, COVID-19 Task Force, federal government, social media, friends and family, and hospitals. The denominator is *N* = 127 for news and faith leader. The denominator is *N* = 126 for coworkers and classmates.

When asked about the degree of trust in different sources of COVID-19 information, the majority trusted doctors the most (69.2%). Just under half of households also had a great deal of trust in COVID-19 information from hospitals, faith leaders, or friends and family (see Fig. 3). Only 36.9% of households had a great deal of trust in information from researchers. Social media (8.5%), drug companies (11.5%), and the news (13.9%) were information sources that the fewest respondents trust a great deal. Among all information sources, social media had the most respondents (36.2%) rate this as information they didn’t trust at all.

## Discussion

These data highlight some important trends within the NHPI community, which continues to be underrepresented (or misrepresented in aggregate data) in research despite facing unique health risks and socioenvironmental contexts. The combined impact of increased risk of exposure to COVID-19 (i.e., traditionally large, multigenerational households, overrepresentation as essential workers) and increased risk of severe outcomes (i.e., immunocompromised or have chronic conditions that are known risk factors) meant NHPIs faced high risks from COVID-19. However, the historic lack of disaggregated public health surveillance data for the NHPI community meant that public health outreach during the pandemic lacked information that described the disproportionate risk facing NHPIs as described above. This meant community members weren’t always able to accurately assess their own personal risk or access needed resources.

Fortunately, community leaders and CBOs helped to fill in the gaps by providing culturally tailored outreach directly to community members (and designing this survey in the absence of available community-specific data). CBOs were able to support community members with critical resources such as meals and groceries, but also emotional, spiritual, and mental health support, and access to COVID-19 resources such as PPE and vaccines. Official public health efforts to provide the public with COVID-19 resources did not adequately reach or cover the NHPI community, possibly due to the lack of data on COVID-19 and NHPIs. For example, the federal government allocated free COVID-19 tests to households in the U.S. (limit of four per household each time) and free N-95 masks at participating pharmacies (up to three), but this was inadequate for NHPI households (average household size of five) where most households included essential workers (at high risk for exposure) and individuals at risk for severe outcomes. This inequity meant NHPI community members had to reach out to other households in search of extra COVID-19 tests in order to keep their household members safe. (T. Lepule, personal communication, April 15, 2021) Since this survey was conducted, local community organizations have also reported a continued demand for culturally relevant resources to support NHPI emotional and mental health.

Despite multiple COVID-19 clinical trials specifically recruiting racial/ethnic minorities within San Diego, only a very small percentage of NHPI households reported that they had participated in a COVID-19 clinical trial and only 9.2% of NHPI households were very likely to participate in a COVID-19 clinical trial. In comparison, a 2020-21 study of mostly Black residents of Louisiana found 10.0% (federally qualified health center patients) and 22.2% (community members) were very willing to participate in a COVID-19 clinical trial [22]. This requires further study to better understand NHPI community perceptions of clinical trials and the risks and benefits of participation, and as one team proposes, the effect of culturally tailored outreach materials [23]. Another study found that only 4 of the 10 clinical trials on the top-selling drug products had disaggregated data on NHPI participation [24]. Even though NHPIs have higher risks for health conditions treated by those drug products, NHPIs were relatively underrepresented in those clinical trials [24]. It may be related to what appears to be less trust in COVID-19 information from researchers compared to other information sources, such as doctors, hospitals, faith leaders, and even friends and family. This lower level of trust may be due to the perceived risks of participation (especially without avenues to ask questions or learn more) or due to a lack of perceived benefits from research (which is further reinforced by a lack of research that is relevant to or representative of NHPIs). In contrast, most households indicated they had already or planned to get the COVID-19 vaccine. The most common concern about getting the vaccine (potential side effects) is similar to study findings from other communities, along with the mistrust of research and the health care system. However, the proportion of vaccinated households in this survey was much lower than local county health departments were reporting, reflecting the ongoing challenge of having quality public health data for NHPIs. To reach NHPI communities more effectively, culturally tailored messaging and culturally relevant strategies may need to be included with any population-level public health communications strategies (especially during public health emergencies). There was low trust in information from the news and social media, which have been the most common ways for mainstream information to be shared at a population-level. Social media has frequently been cited as a source of misinformation, but the lack of trust in the news may indicate larger challenges with mass communication [25]. Despite nearly all households reporting at least one individual who speaks English very well, some households still reported a preference for resources in NHPI languages. We highlight this distinction between language proficiency and language preference as an important one for researchers to understand and be prepared to address.

The relatively higher levels of trust in information from friends and family or faith leaders compared to researchers may be leveraged to help build relationships with researchers to encourage equitable representation in future studies [26–29]. In addition to underrepresentation as research participants, NHPIs are also underrepresented on study teams, especially in leadership roles such as principal investigators. One of the strengths of this study is its community-led process, from survey design to participant outreach. This may be why responses are relatively complete (for example, nonresponse is a known challenge in collecting demographic data in surveys, but this survey had only one missing response to ethnicity). Community members may have been more willing to participate in this type of research because the team leading the research was from the community (with ties to known community leaders and organizations) and there was a clearer potential benefit to their own community.

The data also revealed additional areas that merit further study. For example, while there are high rates of households with immunocompromised individuals or those with chronic conditions, 30% rely on the emergency room or urgent care as a source of regular care. Chronic conditions (such as hypertension or diabetes) require regular, consistent care and check-ups, which means community members may be receiving sub-optimal care for their health. Further research into health care access and barriers to care will be important for addressing these disparities in health outcomes.

### Limitations

One of the limitations of this study is the lack of individual-level data since the questionnaire was designed to collect information at the household level. Due to the nature of this public health emergency and rapid rollout of the questionnaire, the household-level questionnaire was chosen to create less of a burden on families that were already trying to navigate more immediate and critical challenges. Also due to the rapid timeline, the questionnaire was only available in English and online, with the hopes that someone in the household would be able to access the survey. Another limitation is the lack of available contextual information to account for surges in COVID-19 cases or changes in vaccine eligibility/availability or evolution in safety guidelines. These findings are limited to the households that participated in this survey. However, these remain some of the only data available on the direct impact of COVID-19 on the NHPI community.

## Conclusions

Representation of historically marginalized populations in clinical trials is critical for all types of research and not just during a pandemic. When an entire community is not included in public health surveillance data (e.g., lack of reporting of case rates) or when data aren’t reported accurately due to low-quality data (e.g., disaggregated vaccination rates), this can lead to a miscalculation of personal risk at a critical time – and may even be considered another form of misinformation. More precise data could help prevent future inequities in resource distribution (e.g., COVID-19 home tests) and prevent future studies from overlooking important disparities (e.g., age differences in hospitalization risk). For NHPIs, this continued exclusion in research due to lack of community-specific data may be the reason for less trust in researchers, but building intentional partnerships with community leaders and CBOs may be a way forward.

## Supplementary Information

Supplementary Material 1.

## Data Availability

The data generated and analyzed during this study are not publicly available because they belong to the community organization that designed this work, but they are available from the authors upon reasonable request with community permission.

## Abbreviations

CBO: Community-based organization
NHPI: Native Hawaiians and Pacific Islanders
PPE: Personal protective equipment
