# Supplementary material for: Native Hawaiian and Pacific Islander community-led survey on COVID-19 and willingness to participate in clinical trials
Source: BMC Public Health. 2026 Feb 11;26:907. doi: 10.1186/s12889-026-26393-6 (PMC12998361; doi:10.1186/s12889-026-26393-6)
Supplement: Supplementary file 1 — Supplementary Material 1. [file 12889_2026_26393_MOESM1_ESM.pdf]

NHPIs COVID-19 Household Survey - Clinical Trials and Vaccines

As part of COVID-19 (Coronavirus) emergency-response efforts, the National Pacific Islander COVID-19 Response Team and partner organizations are asking you to complete this survey to help us gather information on the current and ongoing needs of our community members and their families.

The data/information collected will help us gain a better understanding of the needs of our community, in order to assist and help policymakers, agencies, and partners address effective culturally responsive measures to meet the needs for our people. Your response is highly needed and greatly appreciated.

As of April 2021, we are going to ask you some questions about COVID-19 clinical trials and vaccines.

- **A clinical trial is a kind of research study. Clinical trials study if treatments or vaccines are safe for people and if they work like they're supposed to.**
- **A vaccine is a substance that helps protect against certain disease.**
- **Right now, clinical trials are being done across the U.S. to see if new treatments and vaccines for COVID-19 work to keep people healthy. Vaccines that have been approved for use are currently being administered to prevent severe COVID-19 disease, hospitalization and death.**

* 1. What is your zip code?

2. If you are in San Diego County, please provide your email address below.

* 3. What is your household’s ethnic group? (check all that apply)

- [ ] Polynesian: Native Hawaiian
- [ ] Polynesian: Samoan
- [ ] Polynesian: Tongan
- [ ] Polynesian: Tahitian
- [ ] Polynesian: Tokelauan
- [ ] Micronesian: Chamorro
- [ ] Micronesian: Marshallese
- [ ] Micronesian: Chuukese
- [ ] Micronesian: Pohnpeian
- [ ] Micronesian: Yapese
- [ ] Micronesian: Kosraen
- [ ] Micronesian: Palauan
- [ ] Micronesian: Carolinian
- [ ] Micronesian: I-Kiribati
- [ ] Melanesian: Fijian
- [ ] Melanesian: Papua New Guinean
- [ ] Melanesian: Solomon Islander
- [ ] Melanesian: Ni-Vanuatu
- [ ] Other Polynesian, Micronesian or Melanesian

* 4. How many people are in your household (living in your house)?

* 5. Number of family members in each age group

0 to 4 years	
5 to 12 years	
13 to 17 years	
18 to 24 years	
25 to 34 years	
35 to 44 years	
45 to 54 years	
55 to 64 years	
65 years or older	

* 6. Do you have at least one adult in your household that speaks English?

- Yes (go to question 7)
- No (go to question 8)

7. How well do they speak English?

- Very well
- Well
- Not well
- I don't know

* 8. For information on health and other services, what language(s) do you prefer? (check all that apply)

- [ ] Samoan
- [ ] Chamorro
- [ ] Tongan
- [ ] Marshallese
- [ ] Chuukese
- [ ] Pohnpeian
- [ ] Palauan
- [ ] Kosraen
- [ ] Yapese
- [ ] Fijian
- [ ] English
- [ ] Other Polynesian, Micronesian or Melanesian

* 9. Is anyone in your household on active duty in the military?

- ( ) Yes
- ( ) No

* 10. Is anyone in your household a US veteran?

- ( ) Yes
- ( ) No

* 11. Do you attend church?

- ( ) Yes
- ( ) No

* 12. Please list the number of people in your household that work in any of these essential occupations. (If no one is working in these occupations, enter "0")

Healthcare or health services (e.g., doctor, medical assistant, home healthcare)

First responder and law enforcement (e.g., police, emergency medical technician, fire fighter)

Food preparation or service (e.g., chef, wait staff)

Sales (e.g., grocery store, gas station, drug store)

Maintenance (e.g., utilities, automotive)

Janitorial/cleaning services

Construction (including plumbing and electrical worker)

Food production or processing (e.g., agriculture, meat processing)

Transit/transportation worker (e.g., bus driver, airport worker)

Teacher, instructor or childcare provider

Protective Services (e.g., security guard)

Community and social services

* 13. Since COVID-19 started, have you or has anyone in your household had to leave your house to go to work?

- Yes
- No

* 14. Did you or members of your household receive Personal Protective Equipment (mask, gloves, etc.) from your employer to prevent the spread of COVID-19?

- Yes
- No
- I don't know

* 15. Has anyone in your household been furloughed, laid off, or had to reduce hours as a result of COVID-19?

- Yes
- No

* 16. Do you or members of your household have a primary care doctor? For example: family doctor, pediatrician, internal medicine doctor.

- Yes (go to question 17)
- No (go to question 18)

17. Including yourself, how many members of your household have a primary care doctor?

* 18. When was the last time you saw a doctor or other health care professional for a physical or regular check-up? Do not include visits when you were sick.

- Never
- Within the past 12 months/year
- 1 to 2 years ago
- 3 to 4 years ago
- 5 to 9 years ago
- 10 years ago or more

* 19. Is there a place that your household usually goes when they are sick?

- Yes
- No
- Don't know

* 20. What kind of place does your household go most often for medical care (select one)?

- Clinic or health center
- Family doctor
- Hospital ER
- Urgent care clinic
- Retail center (for example: CVS, Walgreens)
- There is no one place I go to most often for medical care
- Other

* 21. Do you and your household have any kind of health insurance or health care plan?

- Yes
- No
- Don't know

* 22. How often do you need someone to help you read written information from your doctor or drug store?

- Never
- Rarely
- Sometimes
- Often
- Always

* 23. In your family, check the number of health problems per each individual.

If you have more than FIVE people in your household please choose the top five with the most health problems.

	Person 1	Person 2	Person 3	Person 4	Person 5
High blood pressure	☐	☐	☐	☐	☐
Obesity	☐	☐	☐	☐	☐
Diabetes	☐	☐	☐	☐	☐
Weak immune system	☐	☐	☐	☐	☐
Cancer	☐	☐	☐	☐	☐
Diseases of the lungs (not asthma)	☐	☐	☐	☐	☐
Asthma	☐	☐	☐	☐	☐
Diseases of the heart	☐	☐	☐	☐	☐
Diseases of the kidneys	☐	☐	☐	☐	☐
Diseases of the liver	☐	☐	☐	☐	☐
No health problems	☐	☐	☐	☐	☐
Other (please describe below)	☐	☐	☐	☐	☐

Other, please describe

* 24. Have you or has anyone in your household tested positive for COVID-19?

- ◯ Yes (go to question 25)
- ◯ No (go to question 36)

The following set of questions (#25-35) apply only to people in your household (including yourself if applicable) who tested positive for COVID-19.

* 25. How did you or members of your household find out?

- Clinic or hospital staff
- Health department
- Contact tracer
- Don't know or don't remember
- Other (please specify)

* 26. Were you or members of your household showing any symptoms such as coughing, fever, tiredness, difficulty breathing, muscle aches, loss of smell, taste or any other symptom related to COVID-19?

- Yes (go to question 27)
- No (go to question 28)
- I don't know

27. Including yourself, how many...

went to the clinic, hospital or other health care facility (not emergency room)

went to the emergency room

did not seek health care

* 28. How many needed or wanted to go see a doctor, but did not go or delayed care because they didn't have insurance?

The next questions ask about hospitalization. Please provide information for up to five people who were hospitalized.

* 29. Including yourself, how many were hospitalized?
(If the answer is "0," skip to question 34)

30. Are they hospitalized now?

	Person 1	Person 2	Person 3	Person 4	Person 5
Yes	☐	☐	☐	☐	☐
No	☐	☐	☐	☐	☐

31. How many days were they hospitalized?

	Person 1	Person 2	Person 3	Person 4	Person 5
less than 7 days	☐	☐	☐	☐	☐
8 to 14 days	☐	☐	☐	☐	☐
15 to 21 days	☐	☐	☐	☐	☐
22 to 27 days	☐	☐	☐	☐	☐
28 or more days	☐	☐	☐	☐	☐

32. Are they covered by health insurance?

	Person 1	Person 2	Person 3	Person 4	Person 5
Yes	☐	☐	☐	☐	☐
No	☐	☐	☐	☐	☐

33. Have you or they recovered from COVID-19?

	Person 1	Person 2	Person 3	Person 4	Person 5
Yes	☐	☐	☐	☐	☐
No	☐	☐	☐	☐	☐
I don't know	☐	☐	☐	☐	☐

* 34. How many people in your household have died from COVID-19? (If the answer is "0," skip to question 36)

35. Please select the age of each person in your household (up to 5) who passed away.

	Person 1	Person 2	Person 3	Person 4	Person 5
0 to 4 years	☐	☐	☐	☐	☐
5 to 12 years	☐	☐	☐	☐	☐
13 to 17 years	☐	☐	☐	☐	☐
18 to 24 years	☐	☐	☐	☐	☐
25 to 34 years	☐	☐	☐	☐	☐
35 to 44 years	☐	☐	☐	☐	☐
45 to 54 years	☐	☐	☐	☐	☐
55 to 64 years	☐	☐	☐	☐	☐
65 years or older	☐	☐	☐	☐	☐

NHPIs COVID-19 Household Survey - Clinical Trials and Vaccines

* 36. Have you or members of your household have been isolated or quarantined?

- ◯ Yes (go to next question)
- ◯ No (skip next question)

37. How have you or members of your household been isolated or quarantined at... (check all that apply)

- ☐ Home
- ☐ Relatives/friend's home
- ☐ Church or church housing
- ☐ Hotel/Motel
- ☐ Other (please describe)

* 38. Do you and your household members have access to Personal Protection Equipment (e.g., masks, gloves)?

- ◯ Yes
- ◯ No

* 39. What help (resources or services) do you or anyone in your household need right now because of COVID-19? (check all that apply)

- [ ] Official information in my primary language
- [ ] Food
- [ ] Personal protection equipment like masks, gloves, etc.
- [ ] Housing (Payment for rent/mortgage or protection from eviction)
- [ ] Unemployment
- [ ] Utilities
- [ ] Broadband/Internet
- [ ] Transportation
- [ ] Interpretation services in language for talking to the doctor or other officials
- [ ] Healthcare
- [ ] Mental healthcare
- [ ] Social services (Support with official paperwork like notary, passport, etc.)
- [ ] Insurance coverage (Medicaid, State-based or other insurance coverage)
- [ ] Immigration and other legal concerns
- [ ] School (help with assignments) or childcare for children
- [ ] Elder-care
- [ ] Financial assistance to pay loans or credit cards
- [ ] Small business support
- [ ] Financial assistance to help with COVID-19 related expenses (isolation, quarantine, funeral, etc.)
- [ ] N/A
- [ ] Other (please describe)

* 40. Check 3 biggest problems

- [ ] paying rent, food, etc.
- [ ] caring for sick relatives
- [ ] no space/room for quarantine/isolation
- [ ] paying doctors, hospital bills
- [ ] losing job
- [ ] support or helping kids with school work
- [ ] computer and/or internet access
- [ ] None
- [ ] Other (please describe)

____

* 41. Have you or anyone in your household had any negative interactions with any of the below within the last year? (check all that apply)

- [ ] Law enforcement
- [ ] Healthcare services
- [ ] Schools
- [ ] Housing (e.g., landlord, mortgage lender)
- [ ] Unemployment
- [ ] Social services
- [ ] N/A
- [ ] Other (please describe)

____

* 42. Have you or members of your household registered to vote?

- ○ Yes (go to next question)
- ○ No (skip next question)
- ○ I don't know (skip next question)

43. Including yourself, how many members of your household have registered to vote?

____

* 44. Did you or a member of your household complete the census for 2020?

- Yes
- No
- I don't know

* 45. Has a community-based organization helped you or a member of your household during this pandemic?

Community-based organizations work at the local level to meet community needs. They include social service agencies, nonprofit organizations, and formal and informal community groups, like neighborhood groups or recreational clubs.

- Yes
- No

46. In which areas has the organization(s) helped you during this pandemic? (check all that apply)

- [ ] Meal delivery
- [ ] Financial assistance
- [ ] School-related assistance
- [ ] Emotional/mental health support
- [ ] Childcare
- [ ] Other (please describe)

* 47. Did you or anyone in your household sign up for a COVID-19 clinical trial?

- Yes, I signed up for a clinical trial for a COVID-19 vaccine.
- Yes, I signed up for a clinical trial for a COVID-19 treatment.
- No, I have never signed up for a COVID-19 clinical trial (go to question 49)

48. Where did you or your household member learn how to sign up for a COVID-19 clinical trial (check all that apply; skip to question 51)?

- [ ] COVID-19 Prevention Network (CoVPN)
- [ ] Local county website
- [ ] Social media
- [ ] News (radio, TV, online, newspapers)
- [ ] Community-based organization meeting/town hall/Koviki Talk Podcast
- [ ] Your close friends/family members
- [ ] Your doctor or health care provider
- [ ] Other, please describe

* 49. How likely are you or a household member to sign up for a clinical trial for COVID-19 vaccine or treatment?

1 (Not at all likely)	2	3	4	5 (Very likely)
○	○	○	○	○

* 50. Do you know what to do to sign up for a COVID-19 clinical trial in your area?

○ Yes

○ No

The following questions ask about a COVID-19 vaccine. A vaccine is a substance that helps protect against certain disease.

* 51. What concerns or questions do you or your household have about the COVID-19 vaccine (check all that apply)?

- [ ] Was made too fast/vaccine is too new
- [ ] Mistrust of the research and health care systems
- [ ] Vaccine can give you COVID-19
- [ ] Side effects of vaccine
- [ ] Politicians too involved in development of vaccine
- [ ] Vaccine ingredients
- [ ] Other, please describe

* 52. Have you or anyone in your household received an approved COVID-19 vaccine?

- Yes, I received my 1st vaccine dose
- Yes, I received my 2nd vaccine dose (for Moderna and Pfizer only)
- No (go to question 54)

53. Where did you or your household member learn how to sign up for a COVID-19 vaccine appointment (check all that apply; skip to question 56)?

- [ ] Local county website
- [ ] Social media
- [ ] News (radio, TV, online, newspapers)
- [ ] Your job/employer
- [ ] Community-based organization meeting/ town hall/ Koviki Talk Podcast
- [ ] Your close friends/family members
- [ ] Your doctor or health care provider
- [ ] Other, please describe

* 54. How likely are you and your household to get an approved COVID-19 vaccine when you become eligible?

1 (not at all likely)	2	3	4	5 (very likely)

* 55. Do you know what to do to make an appointment for an approved COVID-19 vaccine in your area once you become eligible?

- Yes
- No

* 56. How much do you and your household trust each of these sources to provide correct information about COVID-19 (prevention, clinical trials, vaccines, etc.)? (Select one response for each row.)

	Not at all	A little	A great deal	Don't know
Your doctor or health care provider	◯	◯	◯	◯
University hospitals	◯	◯	◯	◯
Your faith leader	◯	◯	◯	◯
Your close friends and family members	◯	◯	◯	◯
People you go to work/class with or other people you know	◯	◯	◯	◯
News (radio, TV, online, newspapers)	◯	◯	◯	◯
Social media	◯	◯	◯	◯
The U.S. government	◯	◯	◯	◯
The U.S. Coronavirus Task Force	◯	◯	◯	◯
Companies that make drugs for medical use	◯	◯	◯	◯
People who do research	◯	◯	◯	◯
